# Frequency Properties of Polymer Bonded Compacts Obtained from Ball Milled Permalloy Powders with Mo and Cu Additions

**DOI:** 10.3390/ma16020592

**Published:** 2023-01-07

**Authors:** Florin Popa, Olivier Isnard, Bogdan Viorel Neamțu, Ionel Chicinaș

**Affiliations:** 1 Materials Science and Engineering Department, Technical University of Cluj-Napoca, 103-105 Muncii Avenue, 400641 Cluj-Napoca, Romania; 2 Institut Néel, CNRS/Université de Grenoble Alpes, 25 rue des Martyrs, BP 166, 38042 Grenoble, France

**Keywords:** permalloy, mechanical alloying, nanocrystalline, polymerization, magnetic measurements

## Abstract

Nanocrystalline powders from the Permalloy family, Ni_75_Fe_25_, Ni_79_Fe_16_Mo_5_, and Ni_77_Fe_14_Cu_5_Mo_4_, were obtained by mechanical alloying starting from elemental powders. All compositions were milled for up to 24 h in a high-energy planetary ball mill. The powders were single phase and nanocrystalline as determined by X-ray diffraction studies, with larger flatted particle sizes for Ni_75_Fe_25_ (about 400 μm) and Ni_77_Fe_14_Cu_5_Mo_4_ (about 470 μm), and smaller particle sizes for Ni_79_Fe_16_Mo_5_ (about 170 μm). The homogeneity of the samples was verified by energy-dispersive X-ray spectroscopy (EDX). Soft magnetic composites were obtained by adding 3% of Araldite to the powders, followed by compaction at 700 MPa, and then polymerization. A very good powder covering by the polymer layer was proven by EDX elementals maps. The influence of composition change on the electrical resistivity of the compacts was studied. Hysteresis measurements in static and dynamic fields of up to 10 kHz were recorded, showing the influence of composition and particle size on the compact properties.

## 1. Introduction

The Permalloy alloy family is one of the most-used classes of materials in electrotechnical applications, their development being a constant research topic [1]. This is largely due to their wide magnetic behavior tailored by composition/microstructure and annealing adjustments. One way of developing the Permalloy family consists in obtaining the ternary and quaternary alloys to enhance magnetic properties and extend the frequency use of the alloys to higher frequency ranges [2].

Although binary Permalloys have excellent magnetic properties (high permeability) their use is limited to low frequencies where the eddy currents are small. For higher-frequency applications, a reduction in the electrical conductivity of the materials is required. In the case of Permalloy, this can be achieved by the introduction of nonmagnetic elements such as molybdenum. The introduction of nonmagnetic elements leads to resistivity and, more importantly, permeability increases [3].

Permalloy properties are also found to depend on the production method. Here several techniques both classical and more modern [4] can be mentioned: thin films by sputtering [5], ion beam [6], laser melting [7,8] solid solution precursors [9], additive manufacturing [10,11], soft chemistry [12], spray pyrolysis [13], and mechanical alloying [14,15,16,17,18,19]. 

Of these techniques, mechanical alloying (MA) represents an interesting synthetic method since it implies elemental powder processing in a high-energy ball mill. By milling, stresses are induced in the powders and, by repeated cold welding and fracture events on elemental powder particles, atomic diffusion is promoted between elements, leading to alloy formation [20,21,22]. Mechanical alloying is a technique that allows nanocrystalline materials to be obtained in powder form, which is essential for easy shape forming by powder metallurgy process for industrial applications. 

Nanocrystalline materials are important due to the new/enhanced properties of the classical materials, as in the case of Permalloys. In nanocrystalline soft magnetic materials, the grain boundaries and grain size play a major role, since a decrease with the 6th power of the mean crystallite size is expected for the coercive field [23]. 

Ni-Fe alloys were obtained by mechanical alloying in the whole composition range [24,25,26,27,28,29,30]. In the considered composition range for this study, it was proven that the alloy can be obtained by MA and that properties depend on milling conditions and subsequent annealing [19]. 

Alloying by mechanosynthesis of Ni-Fe compounds is found to be a relatively quick processing action, depending on milling conditions and/or milling equipment (4 h [31], or 8–10 h of milling [32]). The minimum milling time can be reduced by low-temperature annealing. The same milling time range was observed for NiFeMoCu alloy [16]. Supermalloy powders, conversely, needed more time to be formed, an effect induced by the addition of Mo, and the minimum time was around 16 h [33]. The mean crystallite size of the milled powder is an important characteristic, and for the Ni_3_Fe alloy, values of 10–12 nm after 30–52 h of milling were found [17], in the case of NiFeCuMo, a value of 18 nm was found [16]. 

Regarding magnetic properties, nonmagnetic element addition decreases magnetization. Magnetization evolution with milling time differs between materials. For Ni_3_Fe, a relatively significant decrease is observed at shorter milling times, while for NiFeMo a softer decrease is observed in the first hours of milling [34]. 

The product resulting from mechanical alloying is a powder, and to use this powder, a consolidation technique must be chosen. In [4], it was concluded that using MA, the sintering properties of the Ni-Fe powders can be improved by controlling particle agglomeration and improving surface energy, both of which lead to a higher density of compacted samples. 

For the present study, we chose consolidation by pressing and polymerization, leading to the obtaining of soft magnetic composite materials (SMCs). Soft magnetic composite materials are formed by covering the magnetic particles with an insulator layer of polymer. Having such a structure, the material is isotropic and has the advantage of minimizing the eddy currents, due to the polymer insulating layer [35]. In this material class, the nature and amount of insulator is critical, alongside magnetic particle size. When the composite has large particles, a higher permeability is expected, generating better magnetic properties [36,37]. Taken together, it is expected that the applications of soft magnetic material to higher frequencies can be increased [38,39,40]. Although realizing the powder bonding, the polymer is a nonmagnetic material and this will have a consequence on the magnetic properties of the SMC. Some studies showed that if the SMC coating is composed of a resistive magnetic material, higher magnetic properties can be obtained [41,42]. Another important aspect is the compact density and as it was shown in [43] that, as the density increases, better magnetic properties are obtained. 

In this study, we focus on obtaining and comparing the properties of three soft magnetic composites containing (Ni_75_Fe_25_, Ni_79_Fe_16_Mo_5_, and Ni_77_Fe_14_Cu_5_Mo_4_) nanocrystalline powders produced by mechanical alloying after 24 h of milling. This milling time was chosen based on our previous studies on Ni-Fe compositions [14,15,16,17,27]. The consolidation was realized by polymerization, resulting in a nanocomposite toroidal material. The obtained compacts were investigated under AC magnetic fields of up to 10 kHz.

## 2. Materials and Methods

Three alloy compositions (Ni_75_Fe_25_, Ni_79_Fe_16_Mo_5_, and Ni_77_Fe_14_Cu_5_Mo_4_) were obtained by high energy mechanical alloying starting from elemental powders: Ni—carbonyl, Fe—NC100.24, Mo—produced by chemical reduction and Cu powder. The samples were first homogenized for 15 min in a spatial homogenizator and then milled in a homemade planetary ball mill, alongside 59 hardened steel balls. The speed of the main disk was 290 rpm, and the relative speed of the vial was 240 rpm. The powders were introduced in vials filled with argon gas to avoid oxidation. The filling factor of the vial was 60%, and the ball/powder mass ratio was 8:1. The samples were milled for up to 24 h, based on the previous studies conducted on these compositions (more details about the obtaining of the Permalloy alloy by mechanical alloying are given in references [15,16,27]). The powders were then covered by 3 wt.% polymer—Araldite. The covering process can be described as follows: Araldite particles were dissolved in acetone, then the powder was added and mixed until the acetone had evaporated. This mixture was consolidated at 700 MPa in ring shapes and then polymerized in an oven at 150 °C for 3 h.

Structural analysis was performed by X-ray diffraction using a Siemens D 5000 diffractometer (Bruker, Billerica, MA, US); the patterns were recorded in the angular range of 35–103° using Cu Kα radiation (λ = 1.5406 Å). The mean crystallite size and lattice strain were computed using Williamson–Hall plots obtained from the representation of

β* = β·cos(θ)/λ(1)

as a function of

d* = 4·sin(θ)/λ(2)

where β represents the full width at half maximum (FWHM) for the sample, after the subtraction of the instrumental broadening, according to the formula

β = β_meas_ − β_inst_ (3)

where β_meas_ is the FWHM measured in an XRD pattern, and β_inst_ represents the FWHM of an annealed nickel sample used as reference). Using the linear plot fit, from the intercept, the mean crystalline size was calculated, and from the slope, the lattice strain was computed [44,45]. For the computation of the crystallite mean size and lattice strain, Rietveld analysis of the diffraction patterns was performed using the Fullprof software (3.00, version June 2015) [46]. As the instrumental reference, a sample of unmilled nickel powder was used. For estimating the particle size, ImageJ software (1.53t/24 August 2022) was used [47]. 

Hysteresis measurements were performed in DC and AC (up to 10 kHz) conditions on a Remagraph-Remacomp C-705 hysteresisgraph produced by Magnet Physik Dr. Steingroever GmbH (Köln, Germany). 

For the electrical resistivity measurements, an adapted four-probe technique was used. The density was determined using the Archimedes water immersion method. 

The scanning electron microscopy images were recorded using a JEOL JSM 5600 LV (Tokyo, Japan) microscope, and the chemical homogeneity was investigated by energy dispersive X-ray spectroscopy (Oxford Instruments, INCA 200 software, Aztech 4.2 software, High Wycombe, UK).

## 3. Results and Discussion

### 3.1. Mechanical Alloyed Powders

**Figure 1:**
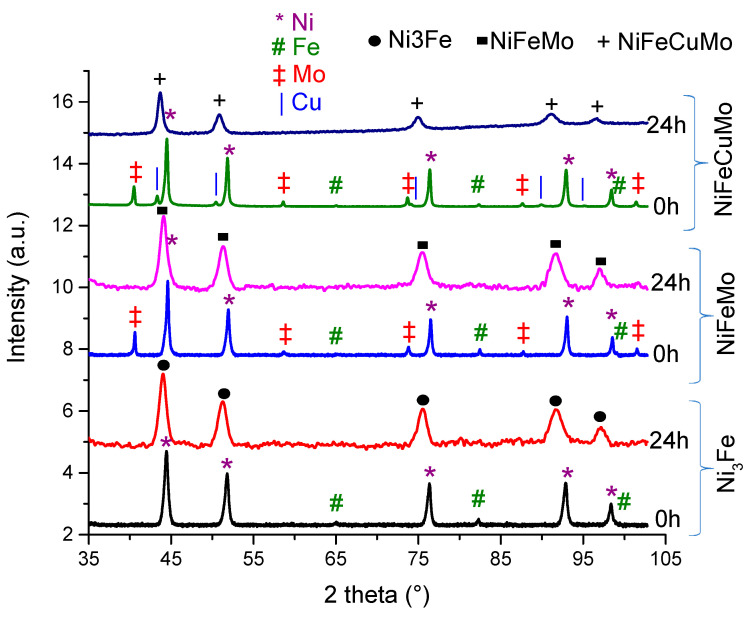
X-ray diffraction patterns for the 24 h milled samples of Ni_75_Fe_25_, Ni_79_Fe_16_Mo_5_, and Ni_77_Fe_14_Cu_5_Mo_4_, respectively.

For all prepared systems, milled for up to 24 h, the recorded X-ray diffraction patterns are presented in Figure 1. For comparison, in each case, the diffraction pattern of an unmilled (0 h) sample is also presented.

All the 0 h samples present only the diffraction peaks of the elements used. After 24 h of milling, in all samples is recorded a single phase, confirming the complete disappearance of elemental Ni, Fe, Mo, and Cu peaks and the formation of the desired phase by mechanical alloying. 

Recorded diffraction patterns for all milled samples present broad peaks, due to small crystallite size and internal stresses induced by milling. Using the Williamson–Hall method, mean crystallite size and internal stress values were computed, and they are presented in Table 1.

Mean crystallite size values indicate the nanocrystalline nature of milled powders, with lower values of mean crystallite size for the Ni_75_Fe_25_ and Ni_79_Fe_16_Mo_5_ samples. For the used experimental conditions, Cu addition to the Ni-Fe alloys (Ni_77_Fe_14_Cu_5_Mo_4_) leads to a higher mean crystallite size. The obtained values for the mean crystallite size are comparable with other studies, but the strain is lower in our case [19,48]. Analyzing the values, a correlation between grain size and lattice strain can be found: smaller grain size samples have smaller lattice strains. This behavior could be related to the fact that the strains induced by milling in the grains can be released by grain fragmentation.

**Figure 2:**
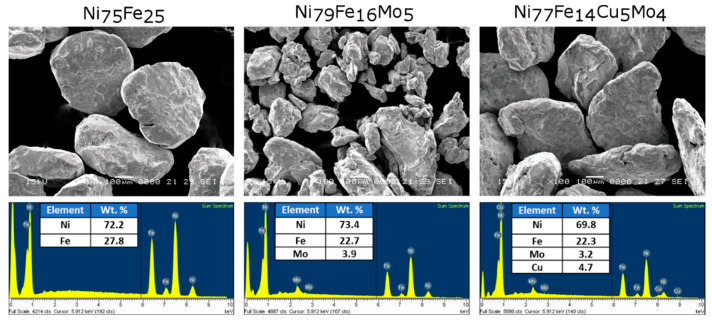
The 24 h milled samples’ morphology and EDX spectra showing the composition.

The influence of the alloying elements on the Ni-Fe alloy morphology is best seen from the SEM images presented in Figure 2. For all samples, the chemical composition is also presented.

In Figure 2, the SEM images show large Ni_75_Fe_25_ particles for the Ni-Fe system. If 5% of Mo is added to the composition, a more brittle character for the powders is induced, leading, after 24 h, to smaller powder particles. Conversely, if Cu and Mo are added, the ductile behavior is promoted, and large particles are again obtained for the Ni_77_Fe_14_Cu_5_Mo_4_ alloy milled for 24 h. Using ImageJ software for analyzing the particle size, we obtained for the Ni_75_Fe_25_ powder, a mean value of about 400 μm, for the Ni_79_Fe_16_Mo_5_ powder, a mean value of about 170 μm, and for the Ni_77_Fe_14_Cu_5_Mo_4_ powder, a mean value of about 470 μm. The larger powders have a flake shape. 

**Figure 3:**
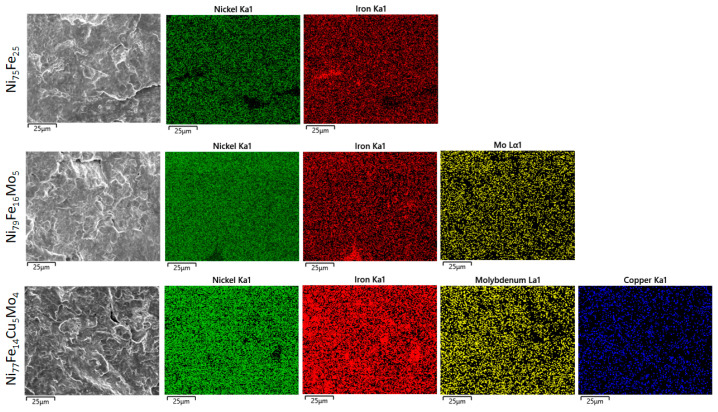
Distribution maps for samples milled for 24 h for the Ni_75_Fe_25_, Ni_79_Fe_16_Mo_5_, and Ni_77_Fe_14_Cu_5_Mo_4_ alloys.

The chemical analysis obtained by EDX indicates that the milled powders possess the desired composition and also a very good homogeneity, as proved by the elements distribution maps recorded for each element (Figure 3). 

### 3.2. Polymerized Compacts 

**Figure 4:**
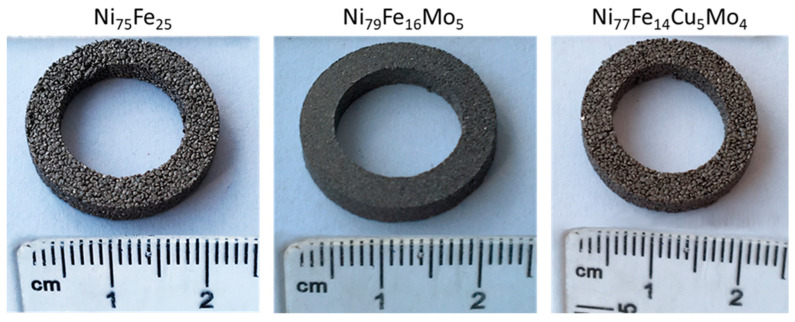
Macroscopic aspect of the toroidal composite compacts obtained from milled powders.

Using the milled powders, compacts were fabricated. The ring sample size and aspect after polymerization are presented in Figure 4. A very smooth surface is obtained for Ni_79_Fe_16_Mo_5_ samples, due to the smaller particle size of the powder.

The electrical resistivities of the toroidal composite compacts were measured and are presented along with their densities in Table 2. In the case of Ni_75_Fe_25_, the electrical resistivity is four orders of magnitude higher than that of a cast sample [4]. The electrical resistivity increase relates to the insulating layer on the individual powder particles and the high amount of grain boundary naturally present in milled powders. As Mo and Cu are added, the electrical resistivity further increases (by a factor of two if only Mo is added, and by a factor of four if both Mo and Cu are added). Although for the cast samples, there is only a small difference between Ni_79_Fe_16_Mo_5_ and Ni_77_Fe_14_Cu_5_Mo_4_, in the case of the milled samples, there is a higher difference. The difference could be the effect of the supplementary disorder induced in the binary alloy by the extra atoms and, as found in reference [49], adding nonmagnetic elements by lowering the long-range order. Electrical resistance increases with the change of the system from binary to ternary were reported also in the case of thin films [6]. 

The density of the composite compacts is similar for the three alloys, with a small increase for the sample containing Cu. The increase, which can be related to Cu ductility, transferred to the alloy, leading to a higher compaction ratio. 

**Figure 5:**
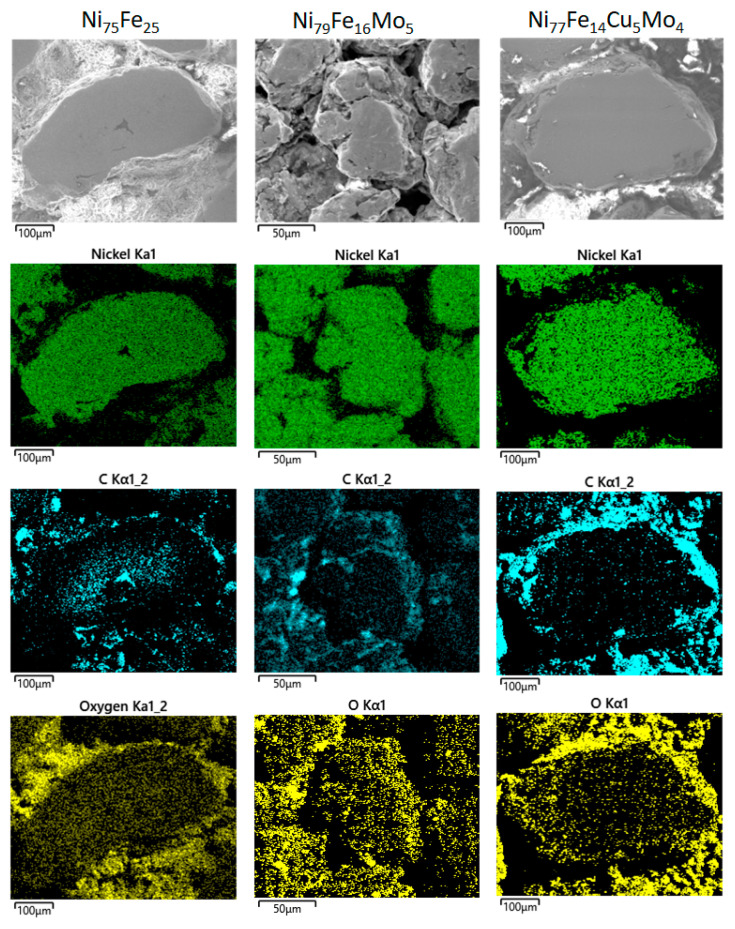
EDX analysis on the compacts, showing the coverage of the metallic particles by the polymer (C and O).

Since the toroidal composite compacts were obtained by polymerization, the covering layer of the particles by polymer was studied by EDX analysis, and the results are shown in Figure 5. In Figure 5, for consistency, are shown only the maps of Ni, and polymer components (C and O).

In Figure 5, the elements C and O are included since they are the representative elements of the polymer used. The C and O distribution maps show the presence of the polymer around alloy particles. For the limitation of eddy current development, an insulating layer is beneficial for the samples. The polymerization of the milled particles, as exemplified by the EDX maps, shows a very good covering, promising good results. 

### 3.3. AC Magnetic Properties of the Composites 

**Figure 6:**
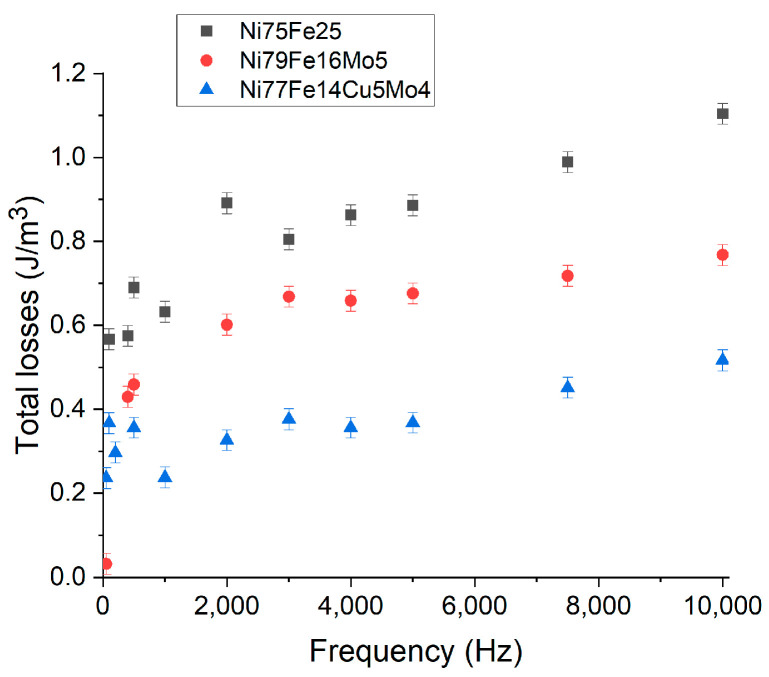
Total losses evolution for Ni_75_Fe_25_, Ni_79_Fe_16_Mo_5,_ and Ni_77_Fe_14_Cu_5_Mo_4_ compacts measured at a field of Bmax = 0.01 T for frequencies up to 10 kHz.

On the above-obtained compacts, AC magnetic properties in frequencies up to 10 kHz were measured. The evolution of the total losses (hysteresis losses and eddy currents) for the three materials at an induction field of 0.01 T are presented in Figure 6. From the total losses, the hysteresis losses can be obtained by extrapolating the evolution of losses at zero frequency, since they do not depend on frequency [51].

The obtained values indicate that smaller core losses are obtained for the higher resistive sample, Ni_77_Fe_14_Cu_5_Mo_4_. A direct comparison between Ni_75_Fe_25_ and Ni_77_Fe_14_Cu_5_Mo_4_ particles shows that an electrical resistivity increase leads to a decrease in overall losses. In addition, if the particles are reduced in size, the losses are reduced further. A similar conclusion was drawn in [39,52]. The lower power losses of the Ni_75_Fe_25_ compacts compared with the values reported in other studies could be due to better insulating layer coverage and particle size control [53].

**Figure 7:**
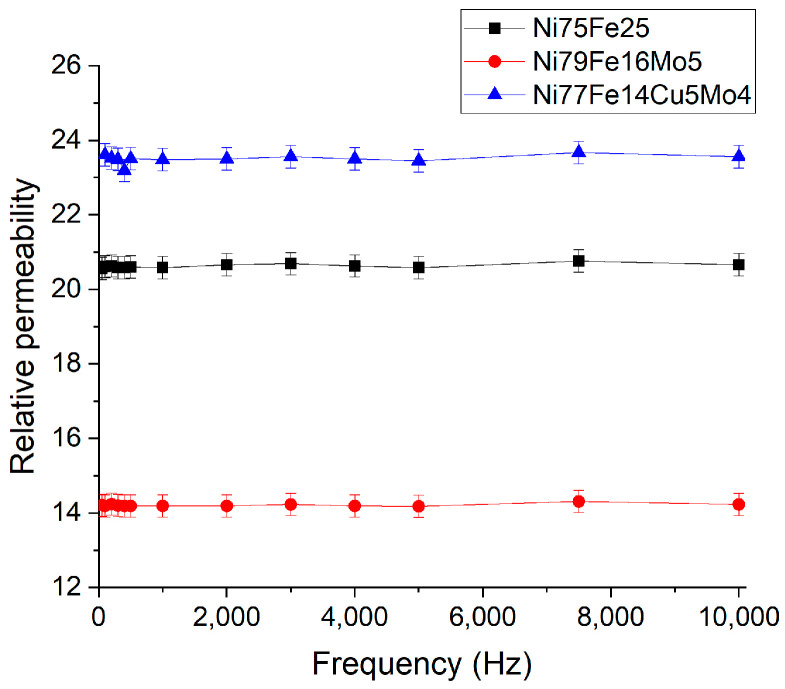
Relative permeability evolution in the frequency range 50 Hz–10 kHz for the Ni_75_Fe_25_, Ni_79_Fe_16_Mo_5,_ and Ni_77_Fe_14_Cu_5_Mo_4_ composite compacts. The applied field was 0.01 T.

Concerning the initial relative permeability versus frequency, there are no visible variations with frequency in the studied range, as can be seen in Figure 7. This indicates that the cutting frequency is higher than 10 kHz, and the possibility of using the compacts in applications at higher frequencies.

The lowest permeability values are obtained for the Ni_79_Fe_16_Mo_5_ sample, probably in connection with the smaller particle size and the polymerization process itself. According to [39], the polymer creates air gaps, and the process is more pronounced for small particles. As shown in Figure 7, the increase in the permeability values from the Ni_75_Fe_25_ compact to the Ni_77_Fe_14_Cu_5_Mo_4_ compact can be attributed to the higher density of the Ni_77_Fe_14_Cu_5_Mo_4_ compact, and, therefore, to a smaller number of air gaps present in the composite compact. Also, it is known that the simultaneous cancelation of the magnetocrystalline anisotropy and the magnetostriction of Ni-Fe alloys is reached by the addition of Cu and Mo. This will also lead to a higher magnetic permeability of the compacts based on Ni_77_Fe_14_Cu_5_Mo_4_ powders. The stability of permeability versus applied frequency is a consequence of the good insulating layer on the surface of the particles [54]. A good insulating layer will hinder the excessive development of eddy currents in the samples and will reduce the skin effect (the penetration depth will be higher) that predominates at higher frequencies. 

**Figure 8:**
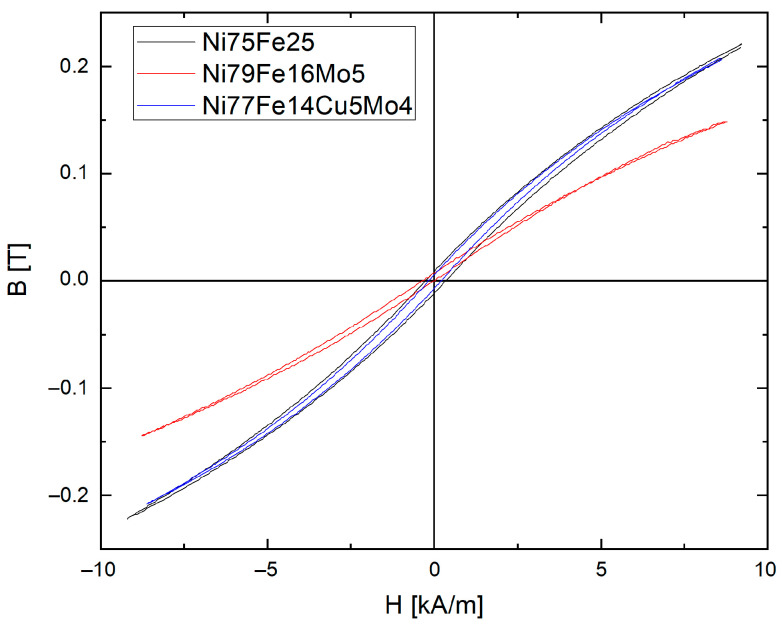
DC hysteresis loops of the toroidal compacts made from Ni_75_Fe_25_, Ni_79_Fe_16_Mo_5,_ and Ni_77_Fe_14_Cu_5_Mo_4_ 24 h milled powders.

DC measurements indicate a shift in the hysteresis tilt, due to the difference in the permeabilities values, as a consequence of larger or smaller particles existing in the sample, as presented in Figure 8.

The hysteresis loops are practically identical for the Ni_75_Fe_25_ and Ni_77_Fe_14_Cu_5_Mo_4_ samples—samples with similar particle size distribution. For the Ni_79_Fe_16_Mo_5_ compact, a more tilted curve is recorded, in connection with smaller particle size. Smaller particle sizes will automatically induce a larger number of air gaps in the compacts and, consequently, larger demagnetizing fields. This will finally lead to a horizontal tilt of the hysteresis loop corresponding to the Ni_79_Fe_16_Mo_5_ compact, indicating a lower magnetic permeability of the compact. A similar conclusion was observed for NiFe-based compacts in [55]. The induction and the coercive field for each compact are given in Table 3.

Thus, the obtained polymerized compacts follow the rule and a large induction is obtained for larger particles and a smaller induction for fine particles [23]. It is worth noting that the samples presented in this study did not reach saturation, as can be observed in Figure 8. However, comparing the induction values of the compacts based on Ni_79_Fe_16_Mo_5_ and Ni_77_Fe_14_Cu_5_Mo_4_ powders, it can be seen that the compact based on Ni_77_Fe_14_Cu_5_Mo_4_ powders presents the largest value for the saturation induction. This is somewhat unusual since the amount of non-magnetic elements is larger in the case of the Ni_77_Fe_14_Cu_5_Mo_4_ powders compared to the Ni_79_Fe_16_Mo_5_ powders. To explain this fact, once again particle size must be considered, and, consequently, the number of air gaps present in the samples. In the case of the compact based on Ni_79_Fe_16_Mo_5_ powders, due to the smaller particle size, a large number of air gaps are created in the composite compact. It is known that air gaps create demagnetizing fields that oppose the magnetizing field. In such a case, even if the magnetizing field is 9 kA/m in both cases (compacts based on Ni_79_Fe_16_Mo_5_ and Ni_77_Fe_14_Cu_5_Mo_4_ powders), the magnetic field experienced by the sample based on Ni_79_Fe_16_Mo_5_ powder is lower leading to a smaller magnetic induction. It seems that the mean crystallite size does not influence magnetic induction. Concerning the coercive field, compacts based on smaller particles have a smaller coercive field. The same explanation concerning the difference that exists between the applied magnetic field and the real field experienced by the sample (difference induced by the air gaps and the demagnetizing fields) can explain the measured values. It is known that the minor hysteresis loops (measured at an induction inferior to the saturation induction) present a coercive field that increases as the induction value approaches saturation induction. As the real value of the applied magnetizing field (the field experienced by the sample) is lower in the case of the sample based on Ni_79_Fe_16_Mo_5_ powders, it is reasonable to expect lower values of the coercive field of this sample. This hypothesis is confirmed, in a way, by the fact that the coercive field of the compacts based on large particles (Ni_75_Fe_25_ and Ni_77_Fe_14_Cu_5_Mo_4_) are practically identical.

## 4. Conclusions

The Permalloy alloys (Ni_75_Fe_25_, Ni_79_Fe_16_Mo_5,_ and Ni_77_Fe_14_Cu_5_Mo_4_) were obtained by mechanical alloying, and after 24 h milling, as single-phase materials with nanocrystalline structure. The obtained powders have a very good chemical homogeneity. By polymerization with 3% Araldite, soft magnetic compacts were obtained.

The electrical resistivity of the compacts is in all cases four times larger than for cast equivalents. As additional elements (Cu and/or Mo) are added to the Ni-Fe powders, the electrical resistivity increases; the quaternary alloy has a four-times larger resistivity than the binary alloy.

In the AC conditions, the lowest power losses and highest permeability are obtained for the compacts with the highest resistivity (Ni_77_Fe_14_Cu_5_Mo_4_). The losses are influenced by the particle size; for smaller particle sizes, the losses are smaller.

The permeability of the samples is dependent on the air-gap content of the samples; as the number of air gaps increases (for small particle sizes), the permeability decreases. Therefore, the Ni_79_Fe_16_Mo_5_ sample has the smallest permeability.

Hysteresis loops recorded in DC condition have a sensitivity toward particle size, leading to a more tilted curve for Ni_79_Fe_16_Mo_5_ samples, with smaller particle sizes. The induction field is more sensitive to the particle size than to mean crystallite size.

## Figures and Tables

**Table 1:** Mean crystallite size and internal stresses for the considered samples milled for 24 h.

Material [Milled 24 h]	Mean Crystallite Size [nm]	Lattice Strain [%]
Ni_75_Fe_25_	19	0.015
Ni_79_Fe_16_Mo_5_	10	0.013
Ni_77_Fe_14_Cu_5_Mo_4_	62	0.041

**Table 2:** Density and electrical resistivity values for the toroidal composite compacts for the Ni_75_Fe_25_, Ni_79_Fe_16_Mo_5,_ and Ni_77_Fe_14_Cu_5_Mo_4_ samples.

Material	Density [g/cm^3^]	Electrical Resistivity [Ω·m]
Ni_75_Fe_25_ (composite compacts)	5.73	1.5 × 10^−3^
Ni_79_Fe_16_Mo_5_ (composite compacts)	5.74	2.5 × 10^−3^
Ni_77_Fe_14_Cu_5_Mo_4_ (composite compacts)	5.94	7.3 × 10^−3^
Ni_75_Fe_25_ (cast)	8.55 [3]	1.6 × 10^−7^ [3]
Ni_79_Fe_16_Mo_5_ (cast)	8.75 [3]	6.0 × 10^−7^ [50]
Ni_77_Fe_14_Cu_5_Mo_4_ (cast)	8.80 [50]	5.8 × 10^−7^ [50]
Ni [50]	8.90	6.9 × 10^−8^
Fe [50]	7.87	1.0 × 10^−7^
Cu [50]	8.96	1.7 × 10^−8^
Mo [50]	10.22	5.7 × 10^−8^

**Table 3:** Density and electrical resistivity values for the toroidal composite compacts for the Ni_75_Fe_25_, Ni_79_Fe_16_Mo_5,_ and Ni_77_Fe_14_Cu_5_Mo_4_ samples.

Material	Induction @ 9 kA/m [T]	Coercive Field [A/m]
Ni_75_Fe_25_	0.23	285
Ni_79_Fe_16_Mo_5_	0.16	135
Ni_77_Fe_14_Cu_5_Mo_4_	0.22	184

## Data Availability

The authors confirm that the data supporting the findings of this study are available within the article.
